# A Rare Presentation of a T-shaped Uterus in the Post-diethylstilbestrol Era: A Case Report and Literature Review

**DOI:** 10.7759/cureus.82950

**Published:** 2025-04-24

**Authors:** Riya Pathare, Omar Abuzeid

**Affiliations:** 1 Obstetrics and Gynecology, Midwestern University Chicago College of Osteopathic Medicine, Chicago, USA; 2 Division of Maternal Fetal-Medicine, Franciscan Physician Network, Crown Point, USA

**Keywords:** diethylstilbestrol, infertility, mullerian uterine anomaly, pregnancy loss, t-shaped uterus, uterine anomaly

## Abstract

The T-shaped uterus is a rare congenital uterine anomaly characterized by a narrowed endometrial cavity with lateral constriction, deviating from the typical triangular shape. Historically, this anomaly has been strongly associated with in-utero exposure to diethylstilbestrol (DES) in the mid-20th century; however, since the discontinuation of DES, no primary cause has been identified. We present the case of a 29-year-old female with a history of recurrent pregnancy loss found to have a T-shaped uterus on sonohysterography. The patient was subsequently referred for surgical evaluation. In patients with recurrent pregnancy loss and nonsignificant inherited and acquired thrombophilia workups, structural uterine abnormalities should be considered in the differential diagnosis. While a T-shaped uterus is associated with infertility and adverse pregnancy outcomes, further research is needed to determine the role of surgical correction in improving reproductive outcomes.

## Introduction

The diagnosis of a T-shaped uterus has been a rare occurrence since the discontinuation of diethylstilbestrol (DES) in 1971. Shortly after DES was discontinued, Kaufman et al. released a study highlighting the presence of a T-shaped uterus in women exposed in utero to DES and this was later followed by the American Fertility Society labeling the T-shaped uterus as a type-VII DES-related Mullerian duct defect [1,2]. More recently, in the Mullerian Anomalies classification (2021), the T-shaped uterus was not recorded as a uterine morphology; however, there have been studies, including the Congenital Uterine Malformation by Experts (CUME) study, to determine more accurate diagnostic criteria for this diagnosis [3]. While rare in the 21st century, the presence of a T-shaped uterus can lead to poorer reproductive outcomes and subfertility, making it an anomaly worth exploring to determine the optimal treatment course. In this study, we detail a case of recurrent pregnancy loss and the eventual discovery of a T-shaped uterus possibly contributing to her poor obstetric outcome. We then discuss diagnostic criteria, treatment, and pregnancy outcomes regarding the T-shaped uterus.

## Case presentation

The patient is a 29-year-old female G2A2, with a history of two first-trimester spontaneous abortions, occurring at 11 weeks and five weeks respectively, without any subsequent dilation and cutterage. The patient presented to her OB/GYN with a history of recurrent pregnancy loss and mild dysmenorrhea in her gynecological history, for which she completed both inherited and acquired thrombophilia labs. The patient had an inherited thrombophilia workup positive for Factor V Leiden heterozygosity and an acquired thrombophilia workup with negative antiphospholipid syndrome labs. Of note, the patient had a positive antinuclear antibody (ANA) without any other definitive positive antibodies, which could be due to a false-positive result. The patient did not have evidence of cervical incompetence, any past history of abdominal trauma, alcoholism, or drug addiction. 

**Figure 1:**
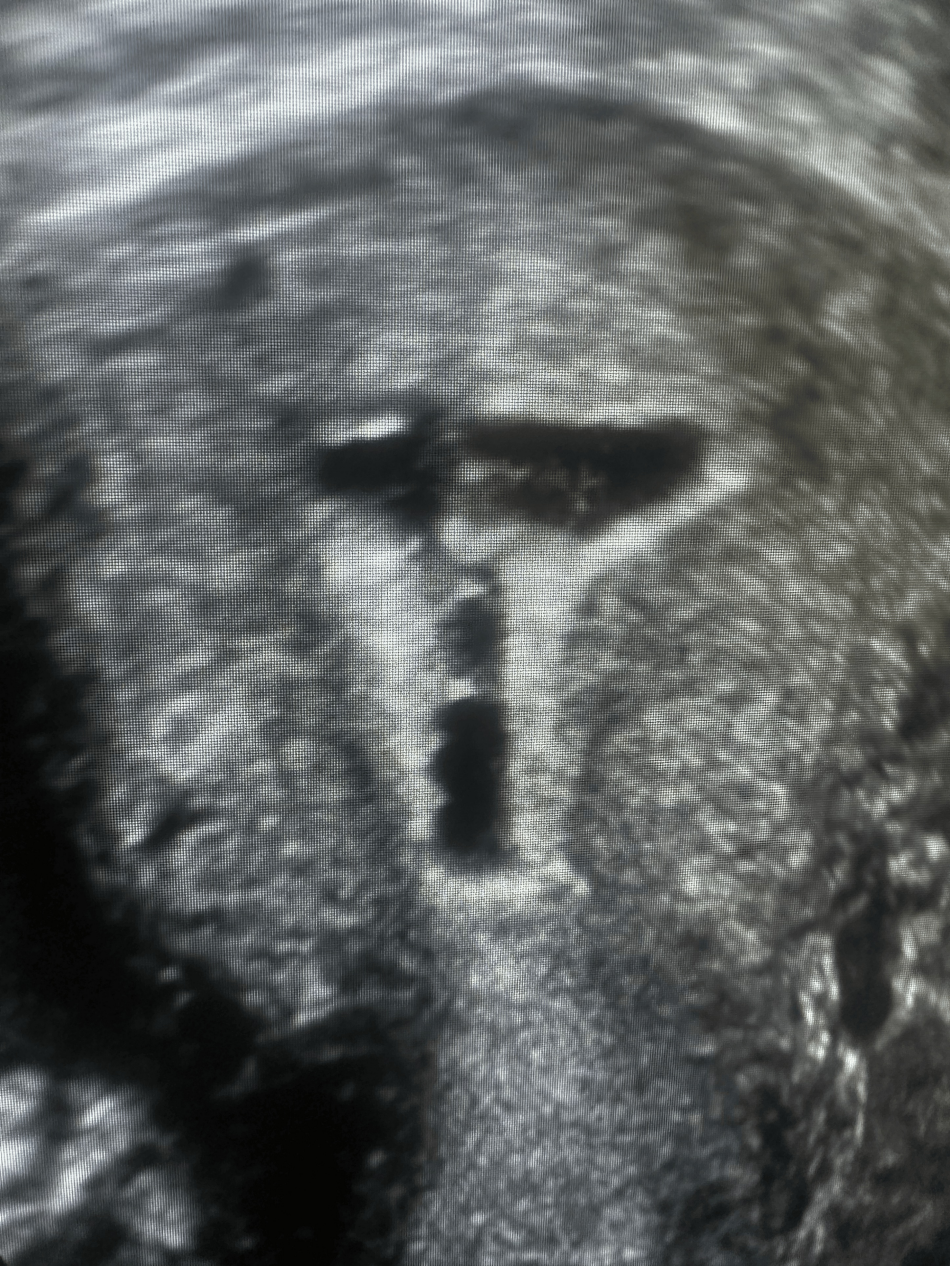
T-Shaped Uterus on Saline Sonohysterogram

She was then referred to Maternal Fetal Medicine for preconception counseling. Maternal and paternal karyotype testing was normal and the patient underwent a 3D saline sonohysterogram to rule out Mullerian anomalies. The procedure was uncomplicated. On the 3D saline sonohysterogram, we discovered a T-shaped uterus and the Mullerian anomaly (Figure 1). Future hysteroscopy and surgical correction were discussed with the patient and appropriate referrals for further testing were provided.

## Discussion

DES was a synthetic estrogen that was prescribed to women between 1940 and 1971 until the Federal Durg Administration advised physicians to stop prescribing the drug in 1971 due to increased incidence of clear cell adenocarcinoma of the cervix and vagina [4,5]. Exposure to DES was also shown to result in infertility, pregnancy complications such as ectopic pregnancy and preterm delivery, and reproductive tract differences such as hooded cervix, pseudopolyps, and a T-shaped uterus [6]. Since the 1980s, due to the declining prevalence of DES, there have been limited studies on the prevalence of T-shaped uteruses in the general population. In 2021, one study of 258 women found a 3.6% prevalence of congenital uterine anomalies as a whole and only a 0.8% prevalence of T-shaped uterus [7]. Still, the prevalence and incidence of T-shaped uteruses remain poorly studied, and no central database has been found for tracking T-shaped uterus cases. Other causes for T-shaped uterus predominate over the exposure to DES in recent times. A systematic review by Coelho et al. reported an equal proportion of secondary causes like adhesions and adenomyosis, for a T-shaped uterus to a primary reason [8]. Secondary causes for T-shaped uterus mentioned above and in-utero exposure to DES were excluded in our patient. There is no common consensus on what may be causing recent cases of T-shaped uterus, and this is likely due to the lack of evaluation of asymptomatic patients in the general population and the classification of the T-shaped uterus as only a minor uterine anomaly [8,9]. However, despite these pitfalls, an accurate diagnosis of a T-shaped uterus should be clarified to provide sufficient care and treatment options for women affected by this diagnosis. 

The earliest and widely accepted classification for a T-shaped uterus comes from the European Society of Human Reproduction and Embryology and the European Society for Gynaecological Endoscopy (ESHRE/ESGE), which define it as a narrow uterine cavity due to thickened lateral walls, with proportions of two-thirds uterine corpus and one-third cervix [10]. More recently, these diagnostic criteria have been called into question given their arbitrary measurement values and the inability to correctly diagnose a T-shaped uterus compared to a normal/arcuate uterus and a uterus infantilis [11]. Given this discrepancy, the CUME released new criteria for diagnosing a T-shaped uterus in 2021. Proper diagnosis of a T-shaped uterus required three measurements, including lateral indentation angle ≤ 130°, lateral indentation depth ≥ 7 mm, and T-angle ≤ 40°, of which none or one of three criteria classified as normal/arcuate uterus, two of three criteria classified as borderline T-shaped uterus, and all three criteria satisfied classified as a T-shaped uterus [11]. A recent 2023 study consisting of 451 women undergoing fertility treatment found that the prevalence of T-shaped uterus through ESHRE/ESGE criteria was 4.1%, while through the CUME criteria, this prevalence reduced to 1.1% [12]. While these guidelines may be strict, they allow for a reduced risk of false positives, which will help determine what may be causing these anomalies after the era of DES and will help tailor diagnostic and treatment options for patients. 

While the T-shaped uterus phenomenon does not pose any risk to the affected patient, it does pose a significant barrier to fertility. When considering diagnostics, most T-shaped uteri have been identified through three-dimensional ultrasound (3D-US); however, other methods have been used to accomplish the same goal. Other methods include magnetic resonance imaging (MRI), hysteroscopy, and saline sonohysterography, which was used in our case study. Additionally, hysteroscopy can be considered for further visualization of the anomaly for additional diagnostics. Multiple options can be considered after diagnosis and when considering past medical history and fertility goals for the patient. Focusing on patients with recurrent miscarriages, like our case, surgical correction can be effective in preventing pregnancy loss. The abnormal T-shaped uterus cavity can be corrected with hysteroscopic meteroplasty, where the myometrium is incised on the lateral wall and fundus to produce the normal triangle-shaped uterine cavity. This has been shown to improve reproductive outcomes in patients with recurrent pregnancy loss, recurrent implantation failure, and long-standing unexplained infertility [13]. As with any surgery, complications included infections, adhesions, and persistence of dysmorphism requiring an additional hysteroscopic approach [8]. While expectant management has been practiced by many physicians who encounter this anomaly, surgery remains a possibility when trying to minimize pregnancy loss. While there has been minimal research on the efficacy of hysteroscopic meteroplasty in the treatment of a T-shaped uterus, this can be offered to patients diagnosed with a T-shaped uterus with a history of infertility or recurrent pregnancy loss to help prevent future pregnancy complications.

## Conclusions

In our case report, we explored an incidence of a T-shaped uterus in a patient with recurrent pregnancy loss, without any genetic anomalies or inherited or acquired thrombophilias. Today’s prevalence, diagnosis, management, and treatment of a T-shaped uterus remain understudied and unclear, with the rare incidence of the abnormality today and the limited information on the general, asymptomatic population. Ultimately, more research must be completed to determine what may be contributing to the prevalence of the T-shaped uterus in this era and what remains the most appropriate and safe treatment options for affected patients.
